# Supplementary material for: Drug-Related Hypersensitivity Reactions Leading to Emergency Department: Original Data and Systematic Review
Source: J Clin Med. 2022 May 16;11(10):2811. doi: 10.3390/jcm11102811 (PMC9143688; doi:10.3390/jcm11102811)
Supplement: Supplementary file 1 [file jcm-11-02811-s001.zip › jcm-1685298-supplementary.pdf]

Supplementary Table S1. MedDRA preferred terms attributable to allergic reactions or anaphylaxis.

pt_code	pt_name	pt_code	pt_name
10068780	Acetate intolerance	10014197	Eczema herpeticum
10056496	Acquired hemophilia with anti FVIII, XI, or XIII	10051890	Eczema impetiginous
10066224	Acute post asthmatic amyotrophy	10014198	Eczema infantile
10001598	Alcohol intolerance	10014199	Eczema infected
10052613	Allergic bronchitis	10014201	Eczema nummular
10059447	Allergic colitis	10066042	Eczema vaccinatum
10053779	Allergic cough	10058681	Eczema vesicular
10051394	Allergic cystitis	10055182	Eczema weeping
10060934	Allergic edema	10056387	Encephalitis allergic
10048594	Allergic granulomatous angiitis	10014627	Encephalopathy allergic
10071198	Allergic hepatitis	10052363	Epstein-Barr virus antigen positive
10057380	Allergic keratitis	10051841	Exposure to allergen
10001715	Allergic myocarditis	10015907	Eye allergy
10061557	Allergic otitis media	10016248	Fat intolerance
10050639	Allergic pharyngitis	10016825	Flushing
10063532	Allergic respiratory disease	10017062	Formication
10063527	Allergic respiratory symptom	10072104	Fructose intolerance
10049153	Allergic sinusitis	10017605	Galactose intolerance
10066173	Allergic transfusion reaction	10059499	Hemorrhagic urticaria
10066536	Allergy prophylaxis	10073224	Hepatitis A antigen
10053462	Allergy test	10063414	Hepatitis B antigen
10056362	Allergy test negative	10071342	Hepatitis B core antibody
10056352	Allergy test positive	10051160	Hepatitis B core antigen
10061626	Allergy to chemicals	10071343	Hepatitis B e antibody
10055048	Allergy to vaccine	10050914	Hepatitis B e antigen
10001890	Alveolitis allergic	10063051	Hepatitis B surface antibody
10063023	Analgesic asthma syndrome	10050529	Hepatitis B surface antigen
10002198	Anaphylactic reaction	10058437	Hepatitis D antigen
10002199	Anaphylactic shock	10058451	Hepatitis D antigen negative
10067113	Anaphylactic transfusion reaction	10058436	Hepatitis D antigen positive
10002216	Anaphylactoid reaction	10057995	Hepatitis E antibody normal
10063119	Anaphylactoid shock	10060028	Hepatitis E antigen

10067010	Anaphylactoid syndrome of pregnancy	10060050	Hepatitis E antigen negative
10049090	Anaphylaxis prophylaxis	10060049	Hepatitis E antigen positive
10002222	Anaphylaxis treatment	10019878	Hereditary fructose intolerance
10002424	Angioedema	10063302	Histamine abnormal
10061443	Antasthmatic drug level	10068652	Histamine intolerance
10061438	Antasthmatic drug level above therapeutic	10060029	Histamine level
10061439	Antasthmatic drug level below therapeutic	10060052	Histamine level decreased
10061442	Antasthmatic drug level therapeutic	10060051	Histamine level increased
10072334	Anti factor X antibody	10063303	Histamine normal
10071464	Anti-actin antibody	10066928	Human antichimeric antibody test
10064059	Antiallergic therapy	10066932	Human anti-human antibody test
10071466	Anti-aquaporin-4 antibody	10066929	Human anti-mouse antibody test
10071467	Anti-basal ganglia antibody	10067432	Human seminal plasma hypersensitivity
10071467	Anti-basal ganglia antibody	10073257	Idiopathic angioedema
10071477	Anti-complement antibody	10021247	Idiopathic urticaria
10076977	Antiendomysial antibody test	10063787	Implant site urticaria
10071462	Anti-epithelial antibody	10049585	Infantile asthma
10071474	Anti-exosome complex antibody	10065490	Infusion site urticaria
10071473	Anti-ganglioside antibody	10058032	Inhibiting antibodies
10063038	Anti-glomerular basement membrane antibody	10066221	Injection site eczema
10071470	Anti-glycyl-tRNA synthetase antibody	10049263	Injection site joint redness
10071469	Anti-IA2 antibody	10022107	Injection site urticaria
10072886	Anti-interferon antibody	10052098	Iodine allergy
10050516<br>10071475	Anti-islet cell antibody<br>Anti-muscle specific kinase antibody	10023681<br>10064866	Lactose intolerance<br>Laryngitis allergic
10071475	Anti-muscle specific kinase antibody	10052331	Leukocyte antigen B-27 positive
10072531	Antimyocardial antibody	10048774	Lymphocytotoxic antibody positive
10071463	Anti-neuronal antibody	10058300	Lysinuric protein intolerance
10050552	Antineutrophil cytoplasmic antibody	10050921	Malaria antibody test
10050894	Anti-neutrophil cytoplasmic antibody positive vasculitis	10070880	Malaria antibody test negative
10071468	Anti-NMDA antibody	10070881	Malaria antibody test positive
10064726	Antinuclear antibody increased	10060066	Measles antibody
10058341	Antiphospholipid antibodies	10068773	Mechanical urticaria
10072533	Antiribosomal P antibody	10071754	Milk soy protein intolerance

10071465	Anti-thrombin antibody	10028164	Multiple allergies
10071472	Anti-transglutaminase antibody	10059638	Mumps antibody test
10071476	Anti-VGCC antibody	10059645	Mumps antibody test negative
10071478	Anti-VGKC antibody	10059644	Mumps antibody test positive
10071461	Anti-vimentin antibody	10029120	Nephritis allergic
10071471	Anti-zinc transporter 8 antibody	10058063	Neutralizing antibodies
10050099	Application site eczema	10064982	Non-neutralizing antibodies positive
10050104	Application site urticaria	10070836	Occupational asthma
10061430	Arthritis allergic	10068355	Oral allergy syndrome
10003553	Asthma	10063927	Orthostatic intolerance
10003559	Asthma late onset	10033993	Parietal cell antibody
10066654	Asthma prophylaxis	10069493	Perennial allergy
10064823	Asthmatic crisis	10063438	Pruritus allergic
10060972	Autoantibody test	10038192	Red man syndrome
10005149	Blepharitis allergic	10039033	Rhesus antibodies
10072187	Breast milk substitute intolerance	10039085	Rhinitis allergic
10006474	Bronchopulmonary aspergillosis allergic	10051126	Scleritis allergic
10071318	Carbohydrate antigen 125 normal	10051401	Small bowler angioedema
10071200	Carbohydrate intolerance	10041961	Status asthmaticus
10007262	Carcinoembryonic antigen	10042432	Sucrose intolerance
10007522	Cardiac asthma	10059212	Tissue polypeptide antigen
10052272	Catheter site urticaria	10072338	Unilateral bronchospasm
10061795	Cerebrospinal fluid measles antibody positive	10046735	Urticaria
10072757	Chronic spontaneous urticaria	10046739	Urticaria aquagenic
10050737	Collagen antigen type 1	10046740	Urticaria cholinergic
10063060	Collagen antigen type IV	10052568	Urticaria chronic
10010744	Conjunctivitis allergic	10046742	Urticaria contact
10010804	Contact lens intolerance	10046750	Urticaria papular
10066973	Contrast media allergy	10046751	Urticaria physical
10012434	Dermatitis allergic	10046752	Urticaria pigmentosa
10068444	Device intolerance	10052572	Urticaria pressure
10013661	Drug allergy	10046755	Urticaria vesiculosa
10061822	Drug intolerance	10069622	Vaccination site urticaria
10013913	Dyshidrotic eczema	10066358	Von Willebrand's factor antibody positive

10014184	Eczema	10068988	Von Willebrand's factor antigen test
10014190	Eczema asteatotic	10068988	Von Willebrand's factor antigen test
10014194	Eczema eyelids		

Supplementary Table S2. Most frequently reported active principles involved in cases of anaphylaxis.

Drugs	N	%
Amoxicillin and beta-lactamase inhibitor	156	25.66
Ceftriaxone	63	10.36
Amoxicillin	56	9.21
Diclofenac	32	5.26
Ketoprofen association	24	3.95
Levofloxacin	20	3.29
Iomeprol	16	2.63
Iopromide	12	1.97
Paracetamol	10	1.64
Acetylsalicylic acid	9	1.48
Ibuprofen	8	1.32
Ciprofloxacin	7	1.15
Paclitaxel	7	1.15
Thiocolchicoside	6	0.99
Ketorolac	5	0.82
Paracetamol, associations excluding psycholeptics	5	0.82
Lansoprazole	5	0.82
Moxifloxacin	5	0.82
Oxaliplatin	5	0.82
Omeprazole	4	0.66
